# Inhibition of hydrogen sulfide biosynthesis sensitizes lung adenocarcinoma to chemotherapeutic drugs by inhibiting mitochondrial DNA repair and suppressing cellular bioenergetics

**DOI:** 10.1038/srep36125

**Published:** 2016-11-03

**Authors:** Bartosz Szczesny, Michela Marcatti, John R. Zatarain, Nadiya Druzhyna, John E. Wiktorowicz, Péter Nagy, Mark R. Hellmich, Csaba Szabo

**Affiliations:** 1 Department of Anesthesiology, University of Texas Medical Branch, Galveston, Texas 77555, USA; 2 Department of Surgery, University of Texas Medical Branch, Galveston, Texas 77555, USA; 3 Department of Biochemistry and Molecular Biology, University of Texas Medical Branch, Galveston, Texas 77555, USA; 4 Department of Molecular Immunology and Toxicology, National Institute of Oncology, Budapest 1122, Hungary

## Abstract

Therapeutic manipulation of the gasotransmitter hydrogen sulfide (H_2_S) has recently been proposed as a novel targeted anticancer approach. Here we show that human lung adenocarcinoma tissue expresses high levels of hydrogen sulfide (H_2_S) producing enzymes, namely, cystathionine beta-synthase (CBS), cystathionine gamma lyase (CSE) and 3-mercaptopyruvate sulfurtransferase (3-MST), in comparison to adjacent lung tissue. In cultured lung adenocarcinoma but not in normal lung epithelial cells elevated H_2_S stimulates mitochondrial DNA repair through sulfhydration of EXOG, which, in turn, promotes mitochondrial DNA repair complex assembly, thereby enhancing mitochondrial DNA repair capacity. In addition, inhibition of H_2_S-producing enzymes suppresses critical bioenergetics parameters in lung adenocarcinoma cells. Together, inhibition of H_2_S-producing enzymes sensitize lung adenocarcinoma cells to chemotherapeutic agents via induction of mitochondrial dysfunction as shown in *in vitro* and *in vivo* models, suggesting a novel mechanism to overcome tumor chemoresistance.

H_2_S is produced in mammalian cells by three distinct enzymes, cystathionine beta-synthase (CBS), cystathionine gamma lyase (CSE) and 3-mercaptopyruvate sulfurtransferase (3-MST); during methionine/transsulfuration pathway^1^,^2^,^3^. Deregulation of either H_2_S production and/or its downstream actions have been implicated in the pathophysiology of several diseases, including cardiovascular disease, shock, inflammation, diabetes, metabolic syndromes and neurodegeneration^4^,^5^,^6^,^7^,^8^,^9^,^10^,^11^. In connection with cancer, we have previously showed a marked increase in the expression of CBS in colorectal cancer cells (compared to the surrounding normal mucosal margin), which was also recapitulated in multiple colon cancer cell lines^12^. ShRNA-mediated silencing, as well as pharmacological inhibition of CBS caused a significant inhibition of the proliferation of colon cancer cells *in vitro* and *in vivo* (in tumor-bearing nude mice). Also, silencing or inhibition of CBS suppressed cellular bioenergetics of the colon cancer cells^12^. The importance of the CBS/H_2_S in the promotion of cell proliferation and cellular bioenergetics has subsequently been confirmed in ovarian cancer^13^ and breast cancer^14^. H_2_S generated by overexpressed CSE, has been implicated in melanoma^15^. In addition a rapidly increasing body of literature implicates the endogenously generated H_2_S to vascular relaxation and angiogenesis, cell proliferation, mitochondrial function, and cell survival - all important factors in cancer biology^16^,^17^,^18^,^19^,^20^,^21^,^22^. However, the mechanisms by which intra-tumor H_2_S maintains cancer cell viability are incompletely understood. Here we investigated unique roles of H_2_S in maintenance of the critical mitochondrial functions involved in chemoresistance of lung adenocarcinoma cells.

## Results

### Lung adenocarcinoma tumors and cultured cells express high level of H_2_S-producing enzymes

**Figure 1:**
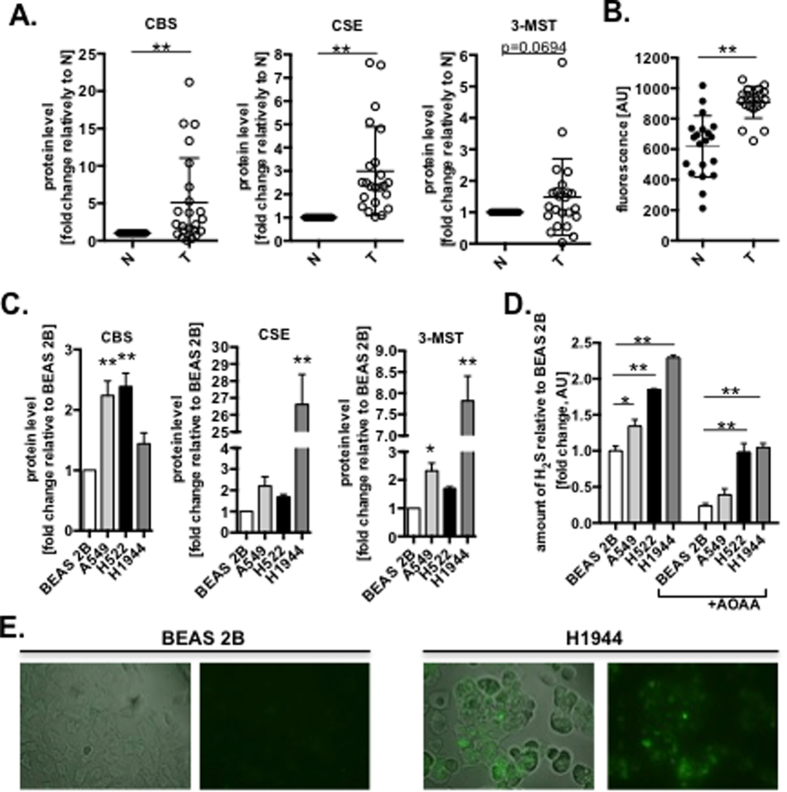
Lung adenocarcinoma cells generate enhanced levels of H_2_S. (**A**) The relative protein level of CBS, CSE and 3-MST and (**B**) production of H_2_S in human lung adenocarcinoma tumors (T, n = 20) and normal adjacent lung tissues (N, set as 1; n = 20) measured by Western blot (WB) analysis and using H_2_S-specific fluorescent probe (AzMC). (**C**) The relative protein level of CBS, CSE and 3-MST analyzed by WB and (**D**) production of H_2_S generated by extracts of normal lung epithelial (BEAS 2B, set as 1) and lung adenocarcinoma (A549, H522, H1944) cells in the absence or present of CBS/CSE inhibitor, AOAA (100 μM). (**E**) The endogenous levels of H_2_S in BEAS 2B and H1944 cells stained with AzMC. Data are mean ± S.E.M. of at least three independent WB analysis and H_2_S detection. Representative images of three independent experiments are shown *P < 0.05, **P < 0.01 (based on two-tailed Student’s *t*-test for pairwise comparison).

We compared human lung adenocarcinoma samples to matched adjacent normal lung tissue and detected significantly higher protein levels of all three known H_2_S-producing enzymes, namely, CBS, CSE and 3-MST (Figs 1A and S1A,B). This was associated with an increased capacity of these tissues to produce H_2_S, as detected by the H_2_S-specific fluorescent probe, 7-azido-4-methylcoumarin (AzMC) (Fig. 1B). Increased expression of the various H_2_S-generating enzymes, and increased H_2_S production was also noted in multiple cultured lung adenocarcinoma cell lines (A549, H522 and H1944), while lung epithelial cells derived from non-malignant tissue (BEAS 2B) showed significantly lower levels of H_2_S production (Figs 1C,D and S1B). H_2_S production was inhibited by aminooxyacetic acid (AOAA), a pharmacological inhibitor of CBS and CSE^23^ (Fig. 1D). The high levels of endogenous H_2_S in lung adenocarcinoma cells was further confirmed by fluorescent live cell imaging (Fig. 1E). As expected from the roles of CBS and CSE in the transsulfuration pathway, AOAA induced the accumulation of serine and homocysteine and decreased cystathionine levels in AOAA-treated A549 cells (Fig. S1C). The remainder of H_2_S production is likely attributed to 3-MST activity (Fig. 1D). Together, these data revealed the high expression of H_2_S-generating enzymes, and increased production of H_2_S in lung adenocarcinoma when compared to normal lung tissue and epithelial cells.

### Repair of the mitochondrial DNA is regulated by H_2_S

**Figure 2:**
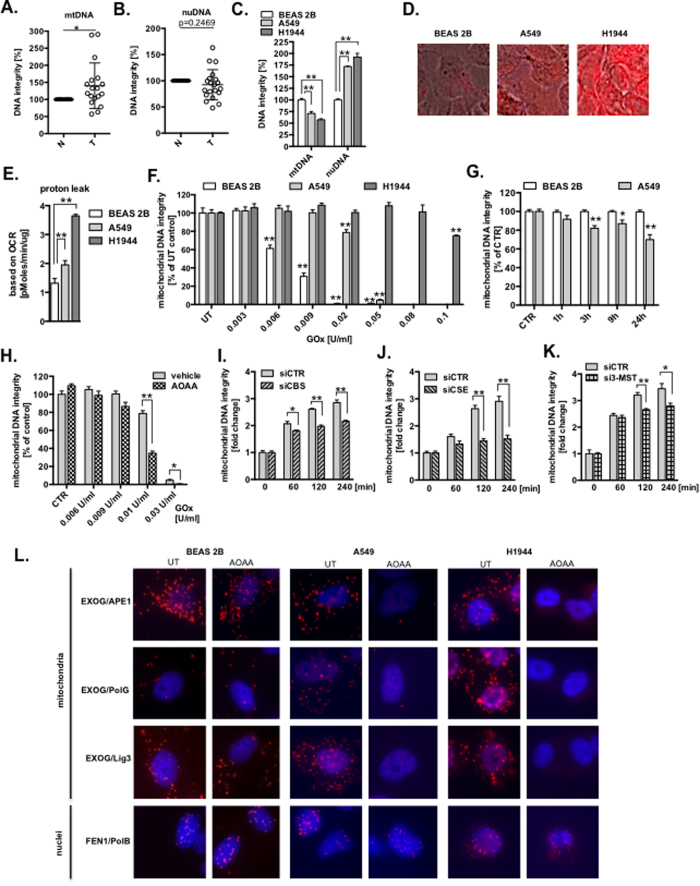
H_2_S mediates enhanced mtDNA integrity in lung adenocarcinoma cells. (**A**,**B**) Comparison of the mitochondrial and nuclear DNA integrity in human lung adenocarcinoma tumors (T, n = 20) and normal adjacent lung tissue (N, set as 100%; n = 20). (**C**) Mitochondrial and nuclear DNA integrity in cultured lung epithelial cells (DNA integrity in normal BEAS 2B cells was set as 100%). (**D,E**) Relative level of oxidative stress measured by MitoSOX Red staining and proton leak-linked to oxygen consumption rate (OCR) profile. (**F**) Integrity of the mtDNA after 1 h challenge with increasing concentration of glucose oxidase (GOx) (DNA integrity in untreated cells was set as 100%). (**G**) MtDNA integrity in cells treated with 1 mM AOAA, an inhibitor of CBS and CSE. (**H**) MtDNA integrity in A549 cells treated with AOAA (1 mM) and GOx for 1 h. (**I**–**K**) Restoration of the mtDNA integrity in A549 cells transiently depleted from CBS, CSE or 3-MST (DNA integrity measured immediately after GOx treatment was set as 1). (**L**) Binary interaction between DNA repair enzymes in the presence or absence of AOAA (300 μM) measured by proximity ligation assay (PLA). Data are mean ± S.E.M of at least three independent analysis of DNA integrity run in technical triplicates. Representative images of three independent experiments are shown *P < 0.05, **P < 0.01 (based on two-tailed Student’s *t*-test for pairwise comparison).

Mitochondrial DNA (mtDNA) encodes an essential set of proteins to maintain oxidative phosphorylation^24^. Although, somatic mutations in mtDNA in various cancers have been suggested to play a role in a selective advantage during oncogenesis^25^,^26^,^27^, a recent study showed that cancer cells with severe mtDNA damage lost tumorigenic potential because of a failure of respiratory functions^28^. Here we compared the DNA integrity in human lung adenocarcinomas with the adjacent lung tissue by the long amplicon PCR assay (LA-qPCR)^29^. Significantly higher integrity of the mtDNA (less DNA breaks) was detected in human lung adenocarcinoma tumors, when compared to matching lung adjacent tissue (Figs 2A and S2A) with no difference in nuclear DNA (Figs 2B and S2B). Higher number of the mtDNA copies was also detected in adenocarcinoma samples (Fig. S2C). Together, we detected more copies of mtDNA in adenocarcinoma tumors with much better preserve mtDNA integrity (less DNA breaks). However, there were more DNA breaks in the mtDNA but less in the nuclear DNA in cultured lung adenocarcinoma cells (A549 and H1944), when compared to normal BEAS 2B cells (Fig. 2C). To solve this discrepancies, we evaluated the extent of intrinsic reactive oxygen species (ROS) production by measuring the level of mt-specific superoxide using MitoSOX; higher ROS signal intensity was observed in A549 and H1944 cells in comparison to BEAS 2B cells (Fig. 2D). This was further supported by observed higher proton leak, a by-product of oxidative phosphorylation, in A549 and H1944, when compared to BEAS 2B cells (Fig. 2E). To assess the role of enhanced endogenous H_2_S biosynthesis on DNA integrity, lung cancer-derived and non-tumor derived epithelial cells were treated with increasing concentration of glucose oxidase (GOx; in the presence of glucose in culture media GOx generates low, continuous levels of hydrogen peroxide). The integrity of the mtDNA was better preserved in GOx-treated A549 and H1944 cells when compared to BEAS 2B (Fig. 2F); nuclear DNA in these conditions was largely unaffected (Fig. S2D). We have recently reported that a mitochondria-targeted H_2_S donor (AP39) protects mtDNA integrity in endothelial cells subjected to oxidative stress^16^,^30^. To test whether endogenous H_2_S production stimulates mtDNA repair in lung adenocarcinoma cells, we monitored the effect of inhibition of CBS/CSE on the integrity of mtDNA and nuclear DNA. The results show that after the pharmacological blockade of tumor H_2_S production, a time-dependent accumulation of DNA damage in the mtDNA (but not in the nuclear DNA) develops in A549 cells (but not in BEAS 2B cells) (Figs 2G and S2E). In addition, inhibition of H_2_S biosynthesis sensitized A549 cells to oxidative mtDNA damage (Fig. 2H). SiRNA-mediated depletion of CSE, CBS, and 3-MST or treatment with CSE-specific inhibitor, D,L-propargylglycine (PAG)^23^, also reduced repair rate of the mtDNA (Figs 2I–K and S2F,G). The oxidative mtDNA damage was prevented by supplementation of A549 cells with mt-specific H_2_S donor, AP39 (Fig. S2H,I). To rule out possible effect of accumulation of serine and homocysteine in cells treated with AOAA on DNA integrity (Fig. S1C), we incubated A549 cells with serine and homocysteine, and measured DNA integrity after 24 h; no significant changes were detected for both genomes (Fig. S2J). Together, our data show that repair of the mtDNA is enhanced by H_2_S.

### H_2_S enhance formation of mitochondrial DNA repair complex

Oxidative stress enhances the formation of DNA repair proficient complexes in the mitochondria^31^. Here, we monitored the binary interactions of the mt-specific DNA repair enzymes EXOG and DNA Polymerase gamma (PolG) and, for comparison, two selected nuclear-specific DNA repair enzymes (FEN1 and PolB)^32^,^33^. The interaction between EXOG with APE1, PolG or Lig3, but not between nuclear-specific DNA repair enzymes FEN1 and PolB, was markedly reduced by H_2_S biosynthesis inhibition in lung adenocarcinoma (but not in normal epithelial cells) (Fig. 2L). To provide direct evidence of the importance of H_2_S in observed integrations between mt-specific DNA repair enzymes; the interaction between EXOG with APE1 or Lig3 was restored by pharmacological replacement of mitochondrial H_2_S in A549 cells treated with AOAA (Fig. S2K). Using mass spectrometry we identified Cys 76 (C76) of EXOG to be post-transnationally modified (sulfhydrated) by oxidized metabolites of H_2_S (Fig. S3A,B). Next, we accessed the interaction of WT EXOG or mutant EXOG (C76A) with APE1, using a co-immunoprecipitation (IP) approach. These studies showed a detectable interaction of WT EXOG with APE1 that was enhanced by treatment with the H_2_S donor, NaHS. In contrast, the interaction of mutant EXOG with APE1 was significantly lower and insensitive to regulation by NaHS (Fig. S3C). Together, our data provide evidences about critical role of H_2_S in formation of mtDNA-specific repair complexes in lung adenocarcinoma cells.

### H_2_S maintain cellular bioenergetics in lung adenocarcinoma cells

**Figure 3:**
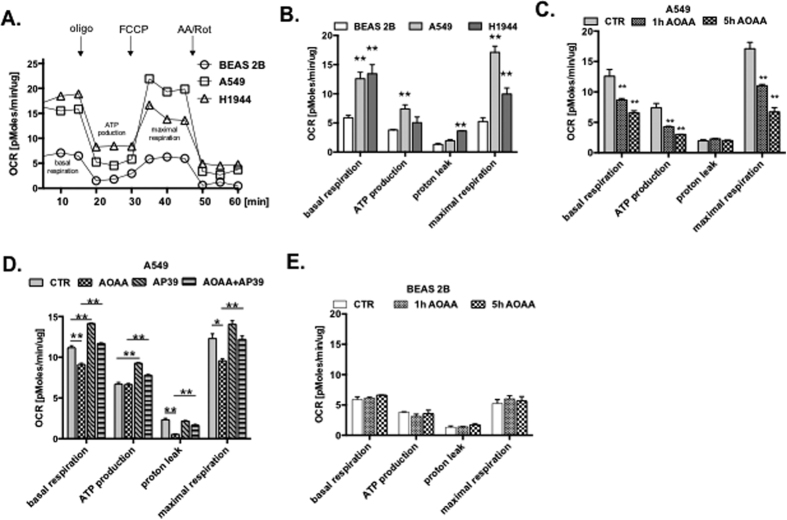
H_2_S is critical for maintaining bioenergetics of lung adenocarcinoma cells. (**A**) Changes in profiles of oxygen consumption rate (OCR) of BEAS 2B, A549 and H1944 cells (oligo; oligomycin, an inhibitor of ATP synthase; FCCP; carbonyl cyanide 4-(trifluoromethoxy)phenylhydrazone, mitochondrial oxidative phosphorylation uncoupler; AA, antimycin A, ROT, rotenone, inhibitors of complex III and I). (**B**) Comparison of cellular bioenergetics parameters of BEAS 2B, A549 and H1944 cells based on OCR. (**C,D**) Time dependent changes in cellular bioenergetics parameters in A549 and BEAS 2B in response to 300 μM AOAA treatment based on OCR. (**E**) Changes in cellular bioenergetics parameters in A549 treated with 300 μM AOAA, 100 nM AP39 or both for 5 h based on OCR. Data are mean ± S.E.M of at least three independent experiments. *P < 0.05, **P < 0.01 (based on two-tailed Student’s *t*-test for pairwise comparison).

Since mtDNA integrity is essential to maintain cellular bioenergetics^34^, we have evaluated the bioenergetic function of cultured lung cells. As expected, higher basal and maximal mitochondrial respiration was detected in A549 and H1944 cells, when compared to BEAS 2B cells (Fig. 3A,B). Moreover, inhibition of H_2_S production reduced all major bioenergetic parameters in A549 cells; these effects were reversed by AP39 (Fig. 3C,D). Importantly, BEAS 2B cell bioenergetics were insensitive to inhibitors of H_2_S production (Fig. 3E). No changes in bioenergetics parameters were detected in A549 cells treated with various concentration of serine and homocysteine (Fig. S3D). From these data, we conclude that lung adenocarcinoma cells are protected from mtDNA damaging agents (e.g., ROS) through the H_2_S-dependent enhancement of mtDNA repair mechanisms, resulting in cancer cell-specific improvements in mitochondrial electron transport and cellular ATP generation.

### Inhibition of H_2_S-producing enzymes sensitize lung adenocarcinoma cells to chemotherapeutic drugs *in vitro* and in tumor bearing mouse model

**Figure 4:**
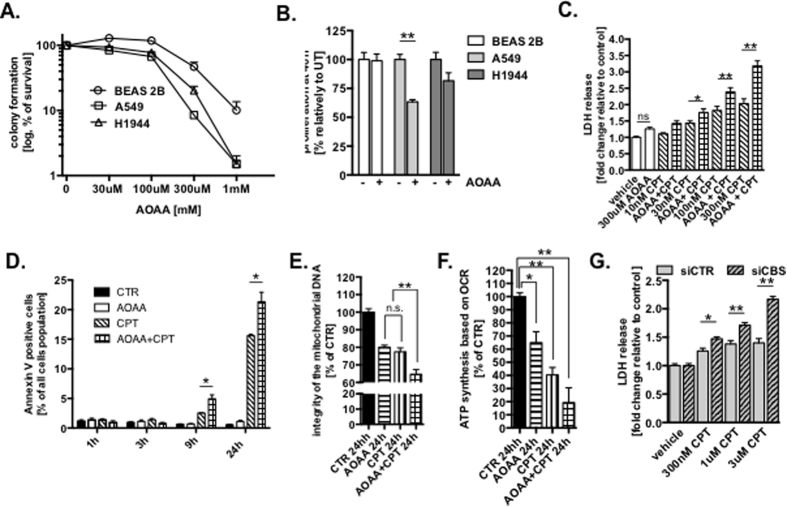
Inhibition of H_2_S production sensitize cultured lung adenocarcinoma cells to chemotherapeutic drugs. (**A**) Viability of BEAS 2B, A549 and H1944 cells to inhibitor of CSE and CBS (AOAA). (**B**) Relative proliferation expressed as % of untreated cells after 48 h of 200 μM of AOAA treatment of cultured lung adenocarcinoma cells. (**C**) Necrotic cell death determined by LDH release of A549 cells treated with AOAA, CPT and AOAA/CPT for 24 h (vehicle treated cells were set as 1). (**D**) Time dependent changes in amount of apoptotic cells measured by Annexin V staining of A549 cells treated with 300 μM AOAA, 300 nM CPT or combination of both. (**E**) MtDNA integrity of A549 cells treated for 24 h with 300 μM AOAA, 300 nM CPT or combination of both. (**F**) Oxygen consumption of A549 cells treated for 24 h with 300 μM AOAA, 300 nM CPT or combination of both. (**G**) Necrotic cell death determined by LDH release of A549 cells transiently transfected with control or CBS-specific siRNA treated with CPT for 24 h (vehicle treated cells were set as 1). Data are mean ± S.E.M of at least three independent analysis of colony formation assay, LDH release, Annexin V staining and of DNA integrity run in technical triplicates. Representative images of three independent experiments are shown *P < 0.05, **P < 0.01 (based on two-tailed Student’s *t*-test for pairwise comparison).

EXOG is essential for mtDNA repair; if its activity is blocked, apoptosis develops^32^,^35^, strongly indicates that accumulation of DNA breaks in mtDNA alone induce cell death. We tested the effect of H_2_S biosynthesis inhibition on the viability of lung cancer cells; A549 and H1944 cells in comparison to BEAS 2B, were significantly less resistant to increasing concentration of AOAA as measured with colony formation assay (Fig. 4A), suggesting essentiality of H_2_S in survival of lung adenocarcinoma cells. Also proliferation of particularly A549 cells was reduced at 48 h in the presence of AOAA (Fig. 4B). We hypothesized that inhibition of H_2_S-generating enzymes, would sensitize lung adenocarcinoma cells to chemotherapeutic drugs. Cellular viability determined by the release of LDH (due to necrotic cell death) showed that although AOAA-treatment alone did not result in decreased viability of either BEAS 2B and A549 cells, in combination with camptothecin (CPT, DNA topoisomerase I inhibitor) a concentration-dependent decrease in viability of A549 (but not BEAS 2B) cells was observed (Figs 4C and S4A). Similarly, treatment with another chemotherapeutic drug, oxaliplatin, in combination with AOAA, resulted in decreased cellular viability of A549 (but not BEAS 2B) cells (Fig. S4B). The combination of AOAA and CPT also resulted in time-dependent, synergistic increase in apoptosis (Fig. 4D), mtDNA damage (Fig. 4E) and decrease oxygen consumption linked with ATP synthesis (Fig. 4F) in A549 cells. In order to provide additional evidence that reduced level of endogenous H_2_S sensitize A549 cells to chemotherapeutic drugs, silencing of CBS also increased LDH release in CPT-treated A549 cells in concentration dependent manner (Fig. 4G).

**Figure 5:**
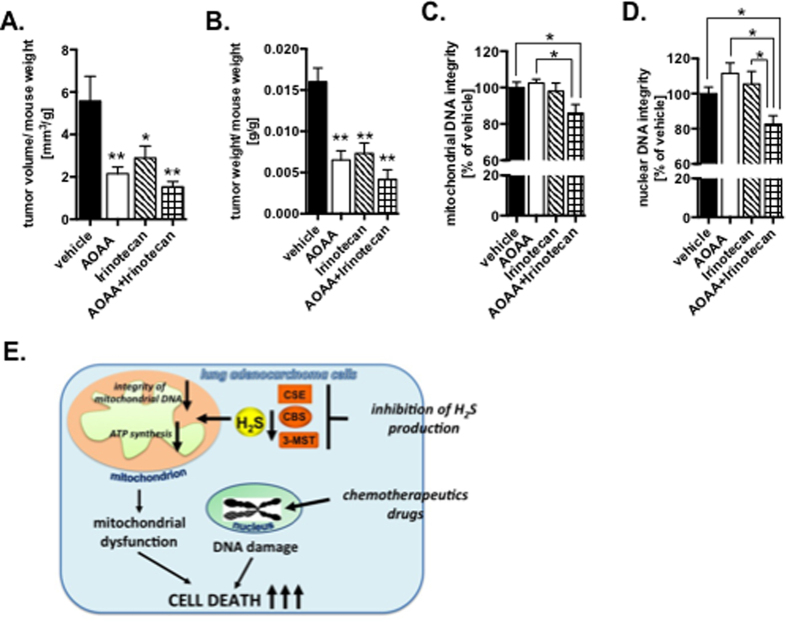
Combination of inhibition of H_2_S-producing enzymes and chemotherapeutic drugs accelerate decreasing of tumor volume in mouse model. (**A,B**) Tumor volumes of mice injected SQ with A549 cells treated with vehicle (PBS), AOAA (3 mg/kg), irinotecan (3 mg/kg) and both (n = 8 for each group). (**C,D**) DNA integrity measured in tumors of the same animals. (**E**) Schematic graph of summary of obtained results. Data are mean ± S.E.M. DNA integrity was run in technical duplicates. *P < 0.05 (based on two-tailed Student’s *t*-test for pairwise comparison).

Finally, we investigated the effect of inhibition of H_2_S biosynthesis in a mouse model of A549 xenografts. Although, tumor volume was reduced in mice treated daily with 9 mg/kg AOAA; its growth resumed when AOAA treatment discontinued and animals were switched to vehicle treatment (Fig. S5A). Next, we reduced the dose of AOAA to 3 mg/kg and investigated the effect of combined treatment with irinotecan. We noted the greatest reduction of tumor growth-rate in animals treated with the combination of AOAA and CPT (Figs 5A,B and S5B,C). Similarly, the most pronounced degree of DNA damage was seen in tumors of mice treated with the AOAA/irinotecan combination (Fig. 5C,D). Comprehensive diagnostic blood profile analysis showed no signs of organ injury in mice treated with the AOAA/irinotecan combination (Table S1).

## Discussion

The above data show that lung adenocarcinoma cells upregulate their H_2_S biosynthesis and use it: (i) to increase the repair capacity of the mtDNA and (ii) to support cellular bioenergetic function; both critical for maintenance of the mitochondrial and cellular homeostasis. Consequently, pharmacological inhibition of H_2_S production in tumor cells induces mitochondrial dysfunction by reduction of DNA repair capacity in the mitochondria, which sensitizes lung adenocarcinoma cells to chemotherapeutic drugs (Fig. 5E). This approach may form the basis of novel combination approaches in the experimental therapy of lung adenocarcinoma and increase therapeutic index of chemotherapeutic drugs, and reduce side effects.

Mammalian cell has hundreds of mitochondria, each containing approximately 10 copies of 16.5 kb plasmid-like mitochondrial DNA (mtDNA)^24^. The mutation rate of human mtDNA is 20- to 100-fold higher that in the nuclear DNA^36^,^37^. One of the reasons is constant challenge of the mtDNA by oxidants generated as a by-product of mitochondrial oxidative phosphorylation. Also, the repair mechanisms of the mtDNA, in contrast to the nuclear DNA, are limited, due to lack of nucleotide excision repair and lack of back-up for the sole DNA polymerase gamma (PolG) and DNA ligase 3; both essential for the correct repair and replication of the mtDNA^33^. This means that the mtDNA is more susceptible to genotoxic agents than the nuclear DNA, as demonstrated by our group and other investigators in various cell types^16^,^35^,^38^,^39^. It is generally accepted that DNA base excision/single strand break repair (BER/SSBR) pathway is the predominant DNA repair mechanism within the mitochondria^33^. There are only two mitochondria-specific BER/SSBR enzymes identified so far: EXOG and PolG^32^,^33^,^34^, the remaining enzymes are shared by the mitochondria and the nucleus.

Mitochondria research, including studies of maintenance of the mtDNA integrity were for a long time neglected in cancer field since upregulation of glycolysis as a major source of energy was proposed, known as a Warburg effect. However, the functional consequence of decreased mtDNA integrity (accumulation of mtDNA damage) for the survival of the cancer cell has been recently re-defined by several studies. In contrast to the nuclear DNA, where DNA instability is one of the hallmark of cancer cells (increase rate of mutations is used by the cancer cell to evade the body’s antitumor mechanisms, and to develop resistance against anticancer agents); with respect to mtDNA, the priority of the cancer cell is to maintain its integrity (i.e. to reduce the amount of DNA breaks)^25^,^26^. It has been recently shown that intact mtDNA is critical for tumorigenic potential^28^. When the integrity of the mtDNA becomes impaired, mitochondrial cell death pathways become activated, the regulation of mitochondrial protein expression becomes impaired, the cellular bioenergetic homeostasis becomes impaired, and the cell’s viability and survivability is suppressed (reviewed in ref. 40. In fact, mtDNA is so critical for the cancer cell, that if the mtDNA is depleted, the cancer cells develop mechanisms to take it up from the host cell, in order to replenish their cellular pools^28^. Some authors even call the mtDNA of the cancer cell “a genetic sanctuary”^41^. Together, various recent studies indicate the importance of the preservation of the mtDNA integrity (meaning proper mtDNA repair); yet the molecular mechanisms of how mtDNA integrity is preserved in cancer cells are poorly defined. Here we are showing for the first time that reduction of the H_2_S-production in lung adenocarcinoma but not in normal lung epithelial cells results in accumulation of the DNA repair intermediates in the mitochondrial but not nuclear DNA. There are only a limited number of published data connecting H_2_S with mtDNA transactions. Using a recently developed mitochondrial-specific H_2_S donor, AP39^30^, we have shown previously that mitochondria-specific H_2_S delivery significantly reduces amount of mtDNA damaged induced by oxidative stress in cultured endothelial cells, suggesting a stimulatory role of H_2_S in process of the mtDNA repair^16^. Another report demonstrated that cells deficient in H_2_S production (due to CSE deficiency), have reduced number of mtDNA copies^42^. Thus - although a correlation between H_2_S and mtDNA damage/repair has been implicated - the molecular mechanisms of how H_2_S regulates the integrity of the mtDNA, and how these processes are important for the tumorigenic potential of cancer cells is currently unknown. Here we showed for the first time that H_2_S directly regulates repair capacity of the mitochondria. Our data indicate that H_2_S supports formation/stabilize mitochondrial DNA repair complexes and thus increases DNA repair capacity within mitochondria. We have shown earlier an active process of formation of the mitochondrial DNA repair complexes in response to oxidative stress^31^. Now we identified critical cysteine residue on EXOG modify by H_2_S which in turn regulates binary interaction with APE1. Both enzymes are critical in DNA end-processing step during BER/SSBR. We showed previously that accumulation of unrepaired DNA intermediates, mostly single strand breaks in the mtDNA induce cell death^32^.

In light of recently emerging data, the role of H_2_S in the regulation of cellular bioenergetics has been also completely re-evaluated. In contrast to the earlier observations demonstrating the toxicity of exogenously added H_2_S as an inhibitor of mitochondrial cytochrome c oxidase (complex IV), recent data indicate that low, physiological concentrations of H_2_S (produced by endogenous, enzymatic sources) has a stimulatory effect on cellular bioenergetics. Physiologically, H_2_S acts as an electron donor and a stimulator of bioenergetics^43^. Hepatoma cells with depleted 3-MST (a constitutive mitochondrial H_2_S-generating enzyme) exhibit reduced bioenergetics parameters^43^. We have shown that nanomolar concentrations of the mitochondrial H_2_S donor, AP39, stimulate cellular bioenergetics in cultured endothelial cells^16^. These data are consistent with a biphasic biological H_2_S dose-response, where many effects of H_2_S at low concentration are beneficial, while at higher concentrations, toxicity develops [reviewed in refs 44 and 45]. However, the role of H_2_S in regulation of bioenergetics in lung cancer cells has not yet been investigated. Our data suggest that enhanced amounts of H_2_S in lung adenocarcinoma cells is also used to maintain cellular bioenergetics.

The enhanced H_2_S biosynthesis demonstrated in the current report selectively supports vital functions of lung adenocarcinoma cells, compared to normal lung epithelial cells. We hypothesized that pharmacological inhibition of H_2_S-generating enzymes or reducing intracellular level of H_2_S by specific scavenger would induce mitochondrial dysfunction which in turn would sensitize lung adenocarcinoma cells towards chemotherapeutic drugs. Our *in vitro* and *in vivo* data confirm that this is a case. Combination of CBS/CSE inhibitor with commonly used chemotherapeutic agents campothecin or oxaliplatin greatly increases cell death of cultured cells and reduced tumor volume in a mouse model. Aminooxyacetic acid (AOAA) - commonly used inhibitor of CBS and CSE - inhibits multiple PLP-dependent enzymes including several oxidoreductases, transferases, hydrolases or isomerases^46^. Therefore, there is a great need to develop novel, more specific inhibitors for H_2_S-producing enzymes, particularly for CSE and CBS, in context of lung adenocarcinoma. Another approach may be to develop mitochondria-specific H_2_S scavengers in order to reduce mitochondria DNA repair capacity and oxidative phosphorylation to induce mitochondrial dysfunction in cancer cells and to sensitize specifically cancer cells to chemotherapeutic drugs.

## Experimental Procedures

### Cell culture

The normal lung epithelial cells BEAS 2B (ATCC CRL-9609; Manassas, VA), and lung adenocarcinoma cells A549 (ATCC CCL-185), NCI-H522 (ATCC CRL-5810) and NCI-H1944 (ATCC CRL-5907), were maintained in RPMI 1640 media supplemented with 10% fetal bovine serum and 100 IU/ml penicillin and 100 μg/ml streptomycin at 37 °C in 5% CO_2_.

### Human lung adenocarcinoma tumors and normal adjacent lung tissue

20 pairs of lung adenocarcinoma tumors and normal adjacent lung tissue was obtained from Lung Cancer Biospecimen Resource Network, University of Virginia Health System, Charlottesville, VA. The experimental protocol was approved by the UTMB Institutional Review Board (IRB), and this study was conducted in compliance with ethical and safe research practices involving human tissues.

### Animal xenograft model of human lung cancer

All animal studies were approved by the Institutional Animal Care and Use Committee (IACUC) of the University of Texas Medical Branch and were carried out in accordance to the approved guidelines. Athymic nude Balb/c male and female mice (8–10 weeks) purchased from Charles Rivers (Wilmington, MA) were injected subcutaneously in either the right or left dorsum with 5–8 × 10^6^ A549 cells as described^12^. 25 or 28 days later, mice were randomized and subcutaneous (SQ) injection of phosphate buffered saline (PBS) as a vehicle, AOAA (3 or 9 mg/kg/day), irinotecan (3 mg/kg) or combination of AOAA/irinotecan (3 mg/kg and 3 mg/kg). Tumor dimensions were measured 2 times per week transcutaneously using a caliper and tumor volume was calculated using the formula π/6 (HxWxL).

### Detection of H_2_S and mitochondrial oxidant production in live cells

40,000 of BEAS 2B, A549 and H1944 cells were seeded in Lab-Tek II chamber coverglass system (Nalgen Nunc International) and incubated at 37 °C and 5% CO_2_ humidified incubator overnight. The cells were loaded with 10 μM of 7-azido-4-methylcoumarin (AzMC, Sigma) for 2 h to detect endogenous H_2_S or with 5 μM of MitoSOX Red dye (Invitrogen) for 10 min to detect mitochondrial superoxide production. Cells were washed three times with PBS and dye’s specific fluorescence was visualized using Nikon eclipse 80i inverted microscope with Photometric CoolSNAP HQ2 camera and NIS-Elements BR 3.10 software.

### Measurement of H_2_S production in cell homogenates

The cultured BEAS 2B, A549, H522 and H1944 cells were washed twice with PBS and homogenized in NP40 lysing buffer (1% NP40, 150 mM NaCl, 50 mM Tris-Cl, pH 8.0) on ice for 30 minutes. The protein concentration determined with Lowry reagent (Bio-Rad) using BSA as a standard of the extract was adjusted to 10 mg/ml. Cell homogenates (20 μl) were incubated in 96-well plates with 5 μM PLP and 2.5 mM of each L-cysteine and homocysteine, and H_2_S production was monitored following fluorescent signal of AzMC (10 μM) at 450 nM for 2 h.

### Cell proliferation

For assessment of cell proliferation, the xCELLigence system (Roche) was used. Briefly, 8,000 of BEAS 2B, A549 or H1944 cells per well were seeded on a E-plate 96, a specially designed 96-well microtiter plate containing interdigitated microelectrodes to non-invasively monitor the cell proliferation by measuring the relative change in the electrical impedance of the cell monolayer. 24 h after initial seeding, cells were treated with 300 μM of AOAA and proliferation monitored for 48 hours.

### Measurement of mitochondrial and nuclear DNA integrity

To measure DNA integrity, we used gene-specific semi-quantitative PCR assays using LongAmp Taq DNA Polymerase (New England BioLabs) as described^29^. Briefly, total DNA was isolated using DNase Blood and Tissue Kit (QIAGEN). Damage to nuclear DNA was estimated by quantification of the PCR amplification of the 10 kb nuclear-specific DNA fragment using PicoGreen fluorescent dye to detect double-stranded DNA (Quant-iT™ PicoGreen, Molecular Probe). Damage to the mitochondrial DNA was estimated by quantification of the PCR amplification of the 8.9 kb mitochondrial-specific DNA fragment using PicoGreen staining. Obtained data was normalized by the PCR amplification of 211 bp mitochondrial genome-specific fragment for correction of the multiple copies of the mitochondrial genome. Preliminary assays were carried out to ensure the linearity of PCR amplification with respect to the number of cycles and DNA concentration. Note, decreased integrity of the DNA mean increasing amount of DNA breaks detected in amplified region.

### Proximity Ligation Assay (PLA)

PLA was performed using Duolink *In situ* Kit (SIGMA) according to the manufacturer’s recommendations with following a pair of primary antibodies raised in two different species: rabbit (r) EXOG (SIGMA), mouse (m) APE1 (Novus Biologicals), m PolG, m Lig3, r FEN1, m PolB (all from GeneTex). Briefly, cells were fixed and permeabilized with 4% paraformaldehyde, 0.2% Tween-20 in PBS for 20 min at room temperature. After blocking for 1 h at room temperature cells were probed with primary antibodies (at 1:200 dilution) overnight at 4 °C. Signal detection and amplification was performed per manufacturer’s instructions. Images were captured using Nikon eclipse 80i inverted fluorescent microscope with Photometric CoolSNAP HQ2 camera and NIS-Elements BR 3.10 software.

### Bioenergetic analysis in cultured cells

The XF24 Extracellular Flux Analyzer (Seahorse Bioscience, Agilent Technologies) was used to measure bioenergetic function. Oxygen consumption rate (OCR) after oligomycin (1.5 μg/ml) was used to assess ATP production rate, OCR after FCCP (0.5 μM) to assess maximal mitochondrial respiratory capacity, OCR after antimycin A (2 μg/ml) and rotenone (2 μM) were used to inhibit the flux of electrons through complex III and I, to detect residual non-mitochondrial oxygen consumption rate, which is considered to be due to cytosolic oxidase enzymes. OCR linked with proton leak was quantified by substation of OCR after antimycin/rotenone from OCR after FCCP. OCR was normalized by μg of protein extract in each well.

### LDH assay

Lactate dehydrogenase (LDH) release was used as a cytotoxicity assay, and as a secondary measurement for determination of necrotic cell death. The changes in absorbance were read kinetically at 492 nm for 15 min on a monochromator-based reader (Powerwave HT, Biotek) at 37 °C. LDH activity values are shown as Vmax for kinetic assays in mOD/min.

### Colony formation assay

200 cells of BEAS 2B, A549 or H1944 cells were seeded on 6 well plates. After 24 h of incubation at 37 °C in 5% CO_2_ media was change to containing various concentration of AOAA. Colonies were counted after 10 days using 1% crystal violet in 50% methanol. Each experiment was repeated 3 times with technical triplicates.

### Determination of apoptotic cell death

Amount of Annexin V positive cells was determined using PE Annexin V Apoptosis Detection Kit (BD Biosciences) according to manufacture’s recommendation. Briefly, A549 cells were treated with 300 μM AOAA, 300 nM CPT or combination of both for 1, 3, 9 and 24 h. Number of Annexin V positive cells was determined using EasyCyte™ Plus Flow Cytometry System (Guava).

### Transient depletion with siRNA

A549 cells were transfected with 40 nM siRNA specific for CBS and 3-MST (#7882, #s194616, Ambion, Life Technologies) and 80 nM siRNA for CSE (#10674, Ambion, Life Technologies) or control siRNA using Lipofectamine 2000 (Invitrogen) per the manufacturer’s protocol. To optimize the siRNA concentration for maximal target depletion, preliminary transfections were carried out with 10–100 nM for each siRNA. The level of depletion was calculated by densitometric analysis of Western blots relatively to loading control of three independent experiments. Cells with 80–90% depletion measured 48 h post-transfection were used in subsequent experiments.

### Statistical analysis

Data are shown as means ± standard error of the mean (SEM). Statistical analysis was performed with Prism G, based on two-tailed Student’s *t*-test for pairwise comparison; *p < 0.05 and **p < 0.01 represent statistically significant differences from control.

## Additional Information

**How to cite this article**: Szczesny, B. *et al*. Inhibition of hydrogen sulfide biosynthesis sensitizes lung adenocarcinoma to chemotherapeutic drugs by inhibiting mitochondrial DNA repair and suppressing cellular bioenergetics. *Sci. Rep.* **6**, 36125; doi: 10.1038/srep36125 (2016).

**Publisher’s note:** Springer Nature remains neutral with regard to jurisdictional claims in published maps and institutional affiliations.

## Supplementary Material

Supplementary Information

## References

[b1] Szabo C. Hydrogen sulphide and its therapeutic potential. Nat Rev Drug Discov 6, 917–935 (2007). 17948022 10.1038/nrd2425

[b2] Kimura H. Hydrogen sulfide: its production, release and functions. Amino acids 41, 113–121 (2011). 20191298 10.1007/s00726-010-0510-x

[b3] Kabil O. & Banerjee R. Enzymology of H_2_S biogenesis, decay and signaling. Antioxid Redox Signal 20, 770–782 (2014). 23600844 10.1089/ars.2013.5339 PMC3910450

[b4] Meng G., Ma Y., Xie L., Ferro A. & Ji Y. Emerging role of hydrogen sulfide in hypertension and related cardiovascular diseases. Br J Pharmacol 172, 5501–5511 (2014). 25204754 10.1111/bph.12900 PMC4667855

[b5] di Masi A. & Ascenzi P. H2S: a “double face” molecule in health and disease. Bio Factors 39, 186–196 (2013). 10.1002/biof.1061 23233276

[b6] Polhemus D. J. & Lefer D. J. Emergence of hydrogen sulfide as an endogenous gaseous signaling molecule in cardiovascular disease. Circ Res 114, 730–737 (2014). 24526678 10.1161/CIRCRESAHA.114.300505 PMC3951140

[b7] Collin M. & Thiemermann C. Hydrogen sulfide and sulfite: novel mediators in the pathophysiology of shock and inflammation. Shock 24, 595–606 (2005). 16317393 10.1097/01.shk.0000188328.59770.25

[b8] Szabo C. Roles of hydrogen sulfide in the pathogenesis of diabetes mellitus and its complications. Antioxid Redox Signal 17, 68–80 (2012). 22149162 10.1089/ars.2011.4451 PMC4701125

[b9] Desai K. M., Chang T., Untereiner A. & Wu L. Hydrogen sulfide and the metabolic syndrome. Expert Rev Clin Pharmacol 4, 63–73 (2011). 22115349 10.1586/ecp.10.133

[b10] Gong Q. H. . A new hope for neurodegeneration: possible role of hydrogen sulfide. J Alzheimers Dis 24, 173–182 (2011). 21441657 10.3233/JAD-2011-110128

[b11] Zhang X. & Bian J. S. Hydrogen sulfide: a neuromodulator and neuroprotectant in the central nervous system. ACS Chem Neurosci 5, 876–883 (2014). 25230373 10.1021/cn500185g

[b12] Szabo C. . Tumor-derived hydrogen sulfide, produced by cystathionine-β-synthase, stimulates bioenergetics, cell proliferation, and angiogenesis in colon cancer. Proc Natl Acad Sci USA 110, 12474–12479 (2013). 23836652 10.1073/pnas.1306241110 PMC3725060

[b13] Bhattacharyya S. . Cystathionine beta-synthase (CBS) contributes to advanced ovarian cancer progression and drug resistance. PLoS one 8, e79167 (2013). 24236104 10.1371/journal.pone.0079167 PMC3827285

[b14] Sen N. . Role of cystathionine β-synthase in human breast cancer. Free Radic Biol Med. 86, 228–38 (2015). 26051168 10.1016/j.freeradbiomed.2015.05.024

[b15] Panza E. . Role of the cystathionine γ lyase/hydrogen sulfide pathway in human melanoma progression. Pigment Cell Melanoma Res 28, 61–72 (2015). 25205294 10.1111/pcmr.12312

[b16] Szczesny B. . AP39, a novel mitochondria-targeted hydrogen sulfide donor, stimulates cellular bioenergetics, exerts cytoprotective effects and protects against the loss of mitochondrial DNA integrity in oxidatively stressed endothelial cells *in vitro*. Nitric oxide 41, 120–130 (2014). 24755204 10.1016/j.niox.2014.04.008 PMC4225488

[b17] Baskar R. & Bian J. Hydrogen sulfide gas has cell growth regulatory role. Eur J Pharmacol 656, 5–9 (2011). 21300051 10.1016/j.ejphar.2011.01.052

[b18] Módis K. . Effect of S-adenosyl-L-methionine (SAM), an allosteric activator of cystathionine-β-synthase (CBS) on colorectal cancer cell proliferation and bioenergetics *in vitro*. Nitric oxide 41, 146–156 (2014). 24667534 10.1016/j.niox.2014.03.001 PMC4156891

[b19] Coletta C. . Hydrogen sulfide and nitric oxide are mutually dependent in the regulation of angiogenesis and endothelium-dependent vasorelaxation. Proc Natl Acad Sci USA 109, 9161–9166 (2012). 22570497 10.1073/pnas.1202916109 PMC3384190

[b20] Szabo C. & Papapetropoulos A. Hydrogen sulphide and angiogenesis: mechanisms and applications. Br J Pharmacol 164, 853–865 (2011). 21198548 10.1111/j.1476-5381.2010.01191.x PMC3195910

[b21] Wang M. J. . The hydrogen sulfide donor NaHS promotes angiogenesis in a rat model of hind limb ischemia. Antioxid Redox Signal 12, 1065–1077 (2010). 19842913 10.1089/ars.2009.2945

[b22] Szabo C. Gasotransmitters in cancer: from pathophysiology to experimental therapy. Nat Rev Drug Discov 15, 185–203 (2016). 26678620 10.1038/nrd.2015.1 PMC5319818

[b23] Asimakopoulou A. . Selectivity of commonly used pharmacological inhibitors for cystathionine β synthase (CBS) and cystathionine γ lyase (CSE). Br J Pharmacol 169, 922–932 (2013). 23488457 10.1111/bph.12171 PMC3687671

[b24] Anderson S. . Sequence and organization of the human mitochondrial genome. Nature 290, 457–465 (1981). 7219534 10.1038/290457a0

[b25] Maybury B. D. Mitochondrial DNA damage is uncommon in cancer but can promote aggressive behaviour. Anticancer Res 33, 3543–3552 (2013). 24023279

[b26] Wallace D. C. Mitochondria and cancer. Nat Rev Cancer 12, 685–698 (2012). 23001348 10.1038/nrc3365 PMC4371788

[b27] Larman T. C. . Spectrum of somatic mitochondrial mutations in five cancers. Proc Natl Acad Sci USA 109, 14087–14091 (2012). 22891333 10.1073/pnas.1211502109 PMC3435197

[b28] Tan A. S. . Mitochondrial genome acquisition restores respiratory function and tumorigenic potential of cancer cells without mitochondrial DNA. Cell Metab 21, 81–94 (2015). 25565207 10.1016/j.cmet.2014.12.003

[b29] Ayala-Torres S., Chen Y., Svoboda T., Rosenblatt J. & Van Houten B. Analysis of gene-specific DNA damage and repair using quantitative polymerase chain reaction. Methods 22, 135–147 (2000). 11020328 10.1006/meth.2000.1054

[b30] La Trionnaire S. . The synthesis and functional evaluation of a mitochondria-targeted hydrogen sulfide donor, (10-oxo-10-(4-(3-thioxo-3H-1,2-dithiol-5-yl)phenoxy) decyl) triphenylphosphonium bromide (AP39). Med Chem Comm 5, 728–736 (2014).

[b31] Szczesny B., Brunyanszki A., Olah G., Mitra S. & Szabo C. Opposing roles of mitochondrial and nuclear PARP1 in the regulation of mitochondrial and nuclear DNA integrity: implications for the regulation of mitochondrial function. Nucleic Acids Res 42, 13161–13173 (2014). 25378300 10.1093/nar/gku1089 PMC4245951

[b32] Tann A. W. . Apoptosis induced by persistent single-strand breaks in mitochondrial genome: critical role of EXOG (5′-EXO/endonuclease) in their repair. J Biol Chem 286, 31975–31983 (2011). 21768646 10.1074/jbc.M110.215715 PMC3173182

[b33] Alexeyev M., Shokolenko I., Wilson G. & LeDoux S. The maintenance of mitochondrial DNA integrity–critical analysis and update. Cold Spring Harb Perspect Biol 5, a012641 (2013). 23637283 10.1101/cshperspect.a012641 PMC3632056

[b34] Kazak L., Reyes A. & Holt I. J. Minimizing the damage: repair pathways keep mitochondrial DNA intact. Nat Rev Mol Cell Biol 13, 659–671 (2012). 22992591 10.1038/nrm3439

[b35] Furda A. M., Marrangoni A. M., Lokshin A. & Van Houten B. Oxidants and not alkylating agents induce rapid mtDNA loss and mitochondrial dysfunction. DNA Repair (Amst) 11, 684–692 (2012). 22766155 10.1016/j.dnarep.2012.06.002 PMC3878289

[b36] Pesole G., Gissi C., De Chirico A. & Saccone C. Nucleotide substitution rate of mammalian mitochondrial genomes. J Mol Evol 48, 427–434 (1999). 10079281 10.1007/pl00006487

[b37] Mandavilli B. S., Santos J. H. & Van Houten B. Mitochondrial DNA repair and aging. Mutat Res 509, 127–151 (2002). 12427535 10.1016/s0027-5107(02)00220-8

[b38] Szczesny B. . Deficiency in repair of the mitochondrial genome sensitizes proliferating myoblasts to oxidative damage. PLoS One 8, e75201 (2013). 24066171 10.1371/journal.pone.0075201 PMC3774773

[b39] Szczesny B., Tann A. W. & Mitra S. Age- and tissue-specific changes in mitochondrial and nuclear DNA base excision repair activity in mice: Susceptibility of skeletal muscles to oxidative injury. Mech Ageing Dev 131, 330–337 (2010). 20363243 10.1016/j.mad.2010.03.009 PMC2883317

[b40] Van Houten B., Hunter S. E. & Meyer J. N. Mitochondrial DNA damage induced autophagy, cell death, and disease. Front Biosci 21, 42–54 (2016). 10.2741/4375 PMC4750375 26709760

[b41] Seoane M. . The mitochondrial genome is a “genetic sanctuary” during the oncogenic process. PLoS One 6, e23327 (2011). 21858071 10.1371/journal.pone.0023327 PMC3157371

[b42] Li S. & Yang G. Hydrogen Sulfide Maintains Mitochondrial DNA Replication via Demethylation of TFAM. Antioxid Redox Signal 23, 630–642 (2015). 25758951 10.1089/ars.2014.6186 PMC4554549

[b43] Módis K., Asimakopoulou A., Coletta C., Papapetropoulos A. & Szabo C. Oxidative stress suppresses the cellular bioenergetic effect of the 3-mercaptopyruvate sulfurtransferase/hydrogen sulfide pathway. Biochem Biophys Res Commun 433, 401–407 (2013). 23537657 10.1016/j.bbrc.2013.02.131

[b44] Szabo C. . Regulation of mitochondrial bioenergetic function by hydrogen sulfide. Part I. Biochemical and physiological mechanisms. Br J Pharmacol 171, 2099–2122 (2014). 23991830 10.1111/bph.12369 PMC3976625

[b45] Módis K. . Regulation of mitochondrial bioenergetic function by hydrogen sulfide. Part II. Pathophysiological and therapeutic aspects. Br J Pharmacol 171, 2123–2146 (2014). 23991749 10.1111/bph.12368 PMC3976626

[b46] Toney M. D. Controlling reaction specificity in pyridoxal phosphate enzymes. Biochim Biophys Acta 1814(11), 1407–18 (2011). 21664990 10.1016/j.bbapap.2011.05.019 PMC3359020

