# Supplementary material for: Inhibition of hydrogen sulfide biosynthesis sensitizes lung adenocarcinoma to chemotherapeutic drugs by inhibiting mitochondrial DNA repair and suppressing cellular bioenergetics
Source: Sci Rep. 2016 Nov 3;6:36125. doi: 10.1038/srep36125 (PMC5093586; doi:10.1038/srep36125)
Supplement: Supplementary Information [file srep36125-s1.pdf]

Supplementary Materials

Inhibition of hydrogen sulfide biosynthesis sensitizes lung adenocarcinoma to chemotherapeutic drugs by inhibiting mitochondrial DNA repair and suppressing cellular bioenergetics.

Bartosz Szczesny[1*], Michela Marcatti[1], John R. Zatarain[2], Nadiya Druzhyna[1], John E. Wiktorowicz[3], Péter Nagy[4], Mark R. Hellmich[2] and Csaba Szabo[1*]

Supplementary Experimental Procedures

Metabolomics analysis

A549 cells in culture were treated with vehicle (PBS), or 1mM AOAA. After incubation for 24 hours, cells were washed with PBS, scraped and snap frozen in liquid nitrogen. Full metabolomic analysis was performed by Metabolon Inc, (Research Triangle Park, NC), as described (48).

Preparation of total cell extract and Western analysis

Total cell extracts were prepared using NP-40 lysis buffer and protein concentration was determined with Lowry reagent (Bio-Rad) using BSA as a standard. Western analysis was performed with the membranes sequentially probed using antibodies against CBS (Abcam, #EPR8579), CSE (Proteintech, #12217-1-AP), 3-MST (SIGMA, #HPA001240), HRP-conjugated actin (Santa Cruz, sc-1616), tubulin (Cell Signaling, #2144), EXOG (Sigma,

#SAB1401498), APE1 (GeneTex, #GTX107122), HPR-linked anti rabbit or mouse secondary antibody (Cell signaling, #7074S, #7076).

Immunoprecipitation

Immunoprecipitation was performed using Dynabeads His-Tag Isolation and Pulldown Kit (Novex, Life Technologies) according to manufacture's recommendation with recombinant WT APE1 (#TP720082, OriGene) and WT and C76A C-terminal His-Tag EXOG (custom order from GenScript). 100ng of APE1 and EXOG was incubated without and with 100nM NaHS for 1h at 37°C followed by pull down using magnetic beads.

In vitro sulfhydration assay

For S-sulfhydration assay, recombinant EXOG (1µg) was incubated with 5mM DTT for 1 h at 37°C, follow up with addition of 10 µM NaHS for 1 h at 37°C. Next, 20mM iodoacetamide was added and samples were incubated for 1h at 37 ^{o}C in dark. Equal volume of 2xSB (without b-ME) was added and samples were run on NuPAGE 4-12% gel. Band corresponding to EXOG was excised and carboxyamidomethyl-S-sulfhydration was analyzed by mass spectrometry.

Nano-LC/MS/MS was performed on Thermo Orbitrap Fusion, coupled with a Dionex Ultimat 3000 nanoHPLC with a 40 well standard auto sampler. The samples were injected onto a nanotrap (100 µm i.d. x 1cm, C18 PepMap 100), followed by a C18 reversed-phase home-packed column (SB-C18, ZORBAX, 5 micron from Agilent) at a flow rate of 400 nL/min with 60 min LC gradient (5% ACN, 0.1% FA to 100% ACN, 0.1% FA). Mass spectrometer parameters include the following: spray tip voltage at +2.2 kV, FTMS mode for MS acquisition

of precursor ions (resolution 120,000); ITMS mode for subsequent MS/MS of top 10 precursors selected; same ions were excluded for 15 sec; MS/MS was accomplished via CID.

Data analysis was performed using the MASCOT server by selecting the corresponding database, followed by uploading the .dat files and mzXML files to ProteIQ 2.7 (Premier Biosoft) to quantify ^{18}O to ^{16}O ratios. The selected analytical parameters included: the enzyme as trypsin; maximum missed cleavages = 2; variable modifications including oxidation (M) and carboxyamidomethyl sulfur (C) for sulfhydration of cysteine; ratios using oxidized M were eliminated from the analysis; precursor ion mass tolerance was set at 5 ppm; fragment ion mass tolerance was 0.6 Da. The significance of a protein match is based on peptide expectation values and the numbers of peptide found (>=2). The default significance threshold is $p<0.05$ to achieve an FDR less than 1.0%, therefore, an expectation value of 0.05 was considered to be on this threshold. Protein identifications were accepted if they could be established at greater than 88.0% probability to achieve an FDR less than 1.0%.

Supplementary References

48. Chaudhri, V.K. et al. Metabolic alterations in lung cancer-associated fibroblasts correlated with increased glycolytic metabolism of the tumor. *Mol Cancer Res* **11**, 579-592 (2013).

Supplementary Figures.

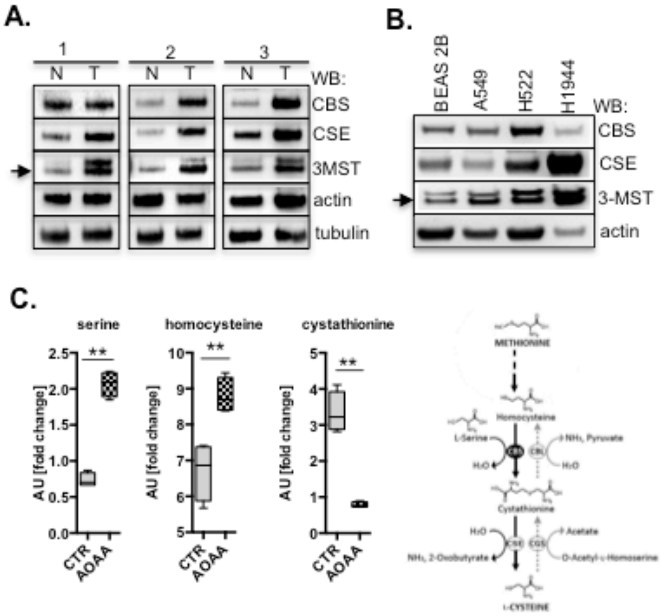
A.
1
2
3
N
T
WB:
CBS
CSE
3MST
actin
tubulin
B.
BEAS 2B
A549
H522
H1944
3-MST
C.
serine
homocysteine
cystathionine
AU [fold change]
CTR
AOAA
**
METHIONINE
Homocysteine
Cystathionine
L-CYSTEINE

Supplementary Figure 1. Lung adenocarcinomas expressed higher level of H_2S-generating enzymes. Western blot of (**A**), three human lung adenocarcinoma (T) and normal adjacent tissues (N) pairs and (**B**), normal lung epithelial (BEAS 2B) and lung adenocarcinoma (A549, H522, H1944) cells. (**C**) Metabolic analysis of transsulfuration pathway of control (CTR) and AOAA (1mM for 24h) treated A549 cells. Schematic representation of the pathway is also shown. Arrow indicates 3-MST specific band. Metabolomic analysis is based on n=4. ** $P<0.01$ (based on two-tailed Student's *t*-test for pairwise comparison).

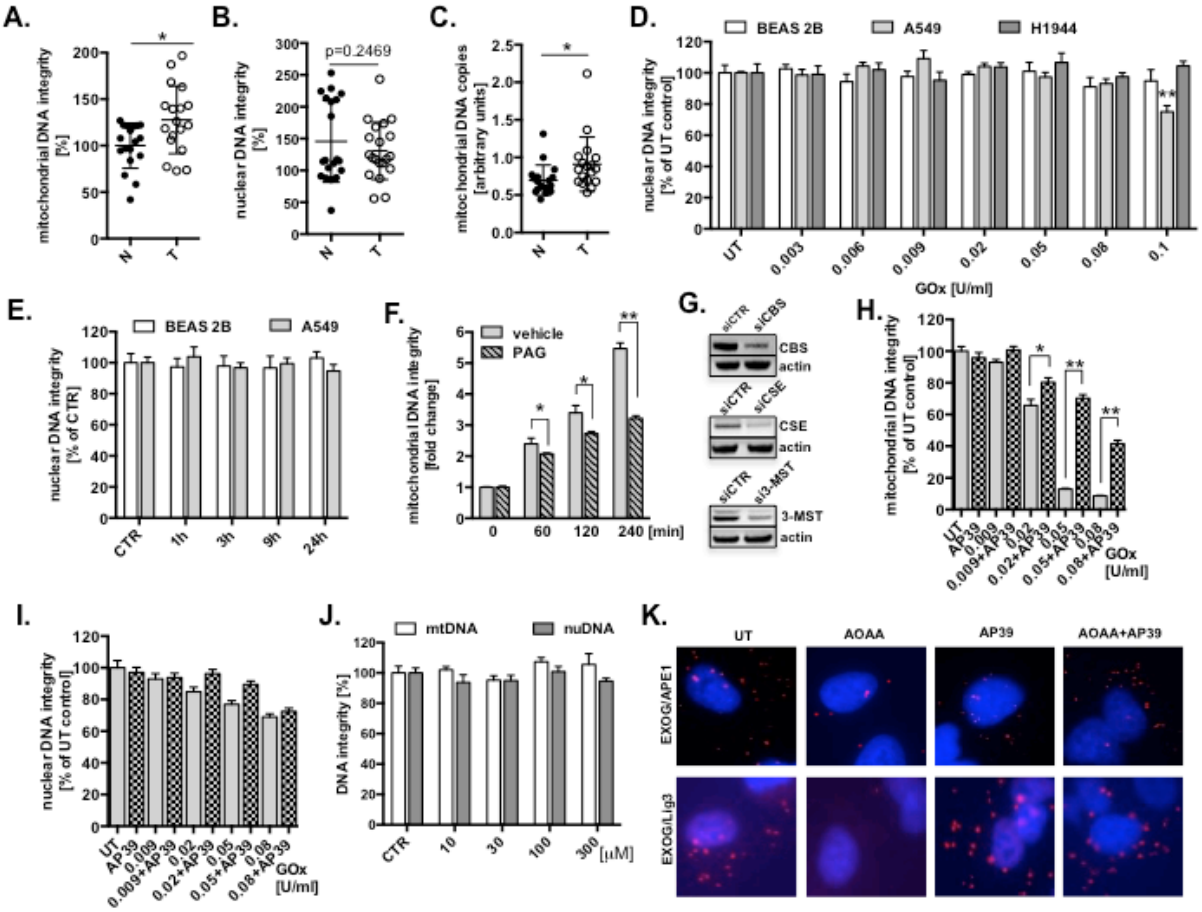
A.
mitochondrial DNA integrity [%]
N
T
B.
nuclear DNA integrity [%]
p=0.2469
C.
mitochondrial DNA copies [arbitrary units]
D.
BEAS 2B
A549
H1944
nuclear DNA integrity [% of UT control]
UT
0.003
0.006
0.009
0.02
0.05
0.08
0.1
GOx [U/ml]
E.
nuclear DNA integrity [% of CTR]
CTR
1h
3h
9h
24h
F.
vehicle
PAG
mitochondrial DNA integrity [fold change]
0
60
120
240 [min]
G.
siCTR
siCBS
CBS
actin
siCSE
CSE
si3-MST
3-MST
H.
mitochondrial DNA integrity [% of UT control]
AP39
0.009+AP39
0.02+AP39
0.05+AP39
0.08+AP39
GOx [U/ml]
I.
nuclear DNA integrity [% of UT control]
J.
mtDNA
nuDNA
DNA integrity [%]
10
30
100
300 [μM]
K.
UT
AOAA
AP39
AOAA+AP39
EXOG/APE1
EXOG/Lig3

Supplementary Figure 2. Repair of the mtDNA is regulated by H_2S. (A, B) Comparison of mitochondrial and nuclear DNA integrity distribution and (**C**) mtDNA copies distribution in human lung adenocarcinoma tumors (T, n=20) and normal adjacent lung tissue (N, n=20). (**D**) Integrity of the nuclear DNA after 1h challenge with increasing concentration of glucose oxidase (GOx) in BEAS 2B, A549 and H1944 cells. (**E**) Nuclear DNA integrity in cells treated with inhibitor of CBS and CSE (1 mM AOAA). (**F**) Restoration of the mtDNA integrity in A549 cells treated with CSE inhibitor (PAG, 3 mM). (**G**) Western blot analysis of CBS, CSE and 3-MST in A549 cells transiently transfected with scramble or targeted siRNA at 48 h post transfection. (**H, I**) Mitochondrial and nuclear DNA integrity of A549 cells treated with 100 nM AP39 (mt-specific H_2S donor) and GOx for 1h. (**J**) Mitochondrial and nuclear DNA integrity of A549 cells treated with 10-300μM of serine and homocysteine for 24h. (**K**) Binary interaction between DNA repair enzymes in the presence of AOAA (300 μM), AP39 (100 nM) or both measured by proximity ligation assay (PLA). Data are mean±S.E.M. of at least three independent analysis of DNA integrity run in technical triplicates. Representative Western blot images of three independent experiments are shown. DNA integrity in untreated cells was set as 100%. * $P<0.05$, ** $P<0.01$ (based on two-tailed Student's *t*-test for pairwise comparison).

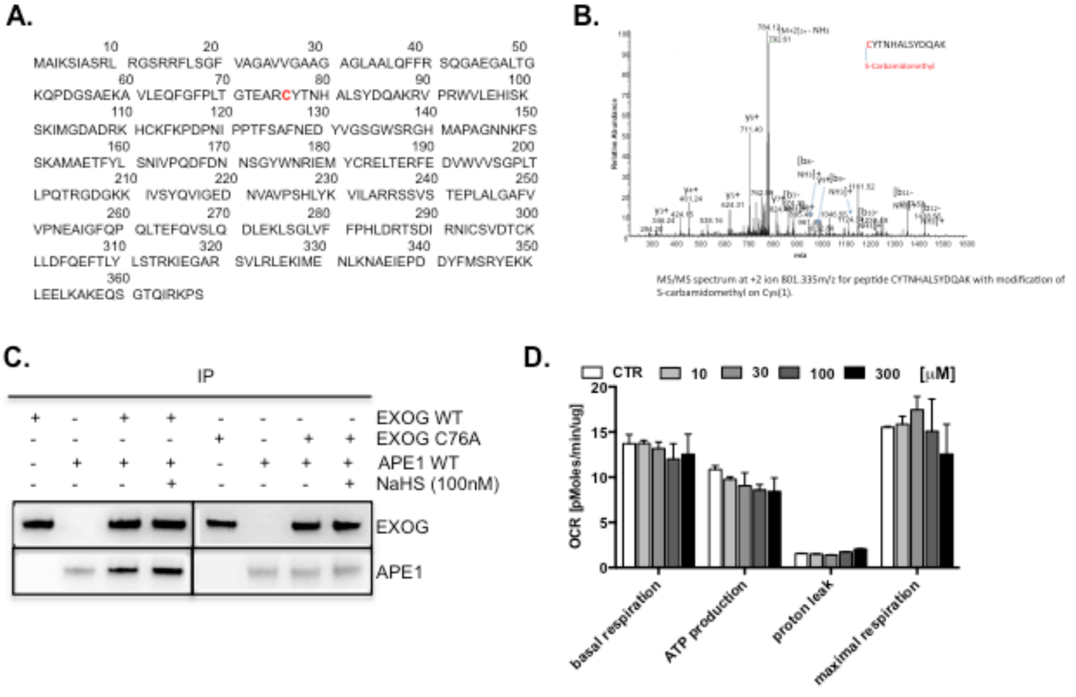
A.
10 20 30 40 50
MAIKSIASRL RGSRRFLSGF VAGAVVGAAG AGLAALQFFR SQGAEGALTG
60 70 80 90 100
KQPDGSAEKA VLEQFGFPLT GTEARCYTNH ALSYDQAKRV PRWVLEHISK
110 120 130 140 150
SKIMGDADRK HCKFKPDPNI PPTFSAFNED YVGSGWSRGH MAPAGNNKFS
160 170 180 190 200
SKAMAETFYL SNIVPQDFDN NSGYWNRIEM YCRELTERFE DVWVVSGPLT
210 220 230 240 250
LPQTRGDGKK IVSYQVIGED NVAVPSHLYK VILARRSSVS TEPLALGAFV
260 270 280 290 300
VPNEAIGFQP QLTEFQVSLQ DLEKLSGLVF FPHLDRTSDI RNICSVDTCK
310 320 330 340 350
LLDFQEFTLY LSTRKIEGAR SVLRLEKIME NLKNAEIEPD DYFMSRYEKK
360
LEELKAKEQS GTQIRKPS
B.
CYTNHALSYDQAK
MS/MS spectrum at +2 ion 801.335m/z for peptide CYTNHALSYDQAK with modification of S-carbamidomethyl on Cys(1).
C.
IP
EXOG WT
EXOG C76A
APE1 WT
NaHS (100nM)
EXOG
APE1
D.
CTR
10
30
100
300
[μM]
OCR [pMoles/min/ug]
basal respiration
ATP production
proton leak
maximal respiration

Supplementary Figure 3. Cysteine 76 of EXOG is modified by H_2S and critical for interaction with APE1. (**A**) Sequence of human EXOG. (**B**) MS/MS spectrum with identified C76 to be modified by NaHS. (**C**) Immunoprecipitation studies of recombinant human WT and C76A EXOG-His-tagged and recombinant WT APE1. Representative Western blot images of three independent experiments are shown. (**D**) Bioenergetics parameters of A549 cells treated with 10-300μM of serine and homocysteine for 24h.

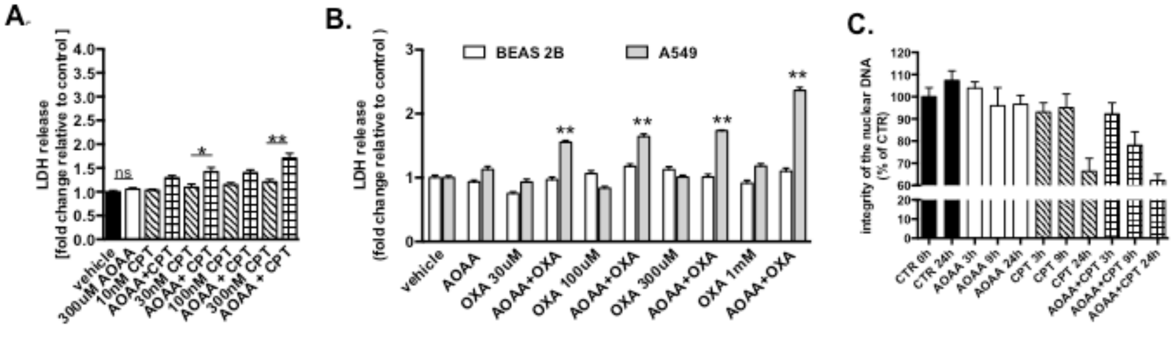

A.
LDH release
[fold change relative to control]
4.0
3.5
3.0
2.5
2.0
1.5
1.0
0.5
0.0
ns
*
**
vehicle
300uM AOAA
10nM CPT
AOAA+CPT
30nM CPT
AOAA+ CPT
100nM CPT
AOAA + CPT
300nM CPT
AOAA + CPT
B.
LDH release
(fold change relative to control)
BEAS 2B
A549
3
2
1
0
**
**
**
**
vehicle
AOAA
OXA 30uM
AOAA+OXA
OXA 100uM
AOAA+OXA
OXA 300uM
AOAA+OXA
OXA 1mM
AOAA+OXA
C.
integrity of the nuclear DNA
(% of CTR)
120
110
100
90
80
70
60
20
10
0
CTR 6h
CTR 24h
AOAA 3h
AOAA 9h
AOAA 24h
CPT 3h
CPT 9h
CPT 24h
AOAA+CPT 3h
AOAA+CPT 9h
AOAA+CPT 24h

Supplementary Figure 4. BEAS 2B cells are less sensitive to combination of AOAA/chemotherapeutic drugs. (**A**) Necrotic cell death determined by LDH release of BEAS 2B cells treated with AOAA, CPT and AOAA/CPT for 24h. (**B**) Comparison of necrotic cell death determined by LDH release of BEAS 2B and A549 cells treated with AOAA (300 μM), oxaliplatin (OXA) and AOAA/OXA for 24h. (**C**) Time dependent changes in nuclear DNA integrity of A549 cells treated with 300 μM AOAA, 300 nM CPT or combination of both. Data are mean±S.E.M. of at least three independent analyses of LDH release and of DNA integrity run in technical triplicates. * P<0.05, ** P<0.01 (based on two-tailed Student's t-test for pairwise comparison).

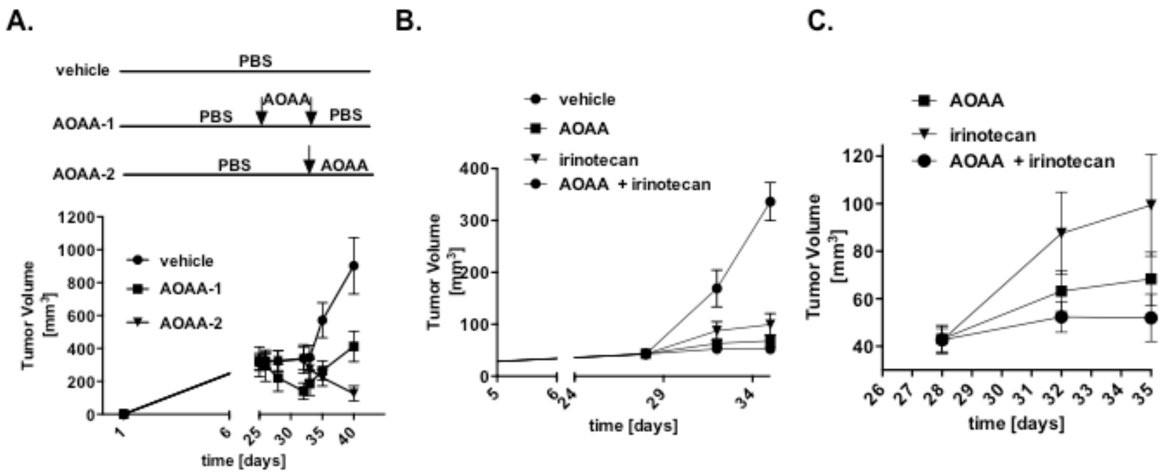
A.
vehicle
PBS
AOAA-1
PBS
AOAA
PBS
AOAA-2
PBS
AOAA
Tumor Volume [mm3]
vehicle
AOAA-1
AOAA-2
time [days]
B.
vehicle
AOAA
irinotecan
AOAA + irinotecan
Tumor Volume [mm3]
time [days]
C.
AOAA
irinotecan
AOAA + irinotecan
Tumor Volume [mm3]
time [days]

Supplementary Figure 5. Analysis of tumor volume in A549 subcutaneous tumor xenografts. (**A**) 25 days post injection of $8x10^6$ A549 cells mice were randomized and treated with vehicle (PBS) for 14 days (vehicle, n=5); 9 mg/kg of AOAA for a 7 days followed 7 days with PBS treatment (AOAA-1, n=5); PBS for a 7 days followed by 7 days of treatment with 9 mg/kg of AOAA (AOAA-2, n=5). (**B, C**) 28 days post injection of $5x10^6$ A549 cells mice were treated for 7 days with PBS (vehicle, n=8), 3mg/kg AOAA (n=8), 3mg/kg irinotecan (n=8) or combination of AOAA and irinotecan (n=8). Data are mean±S.E.M.

		RANGE	VEHICLE 1 average	sd	AOAA4 average	sd	IRINOTECAN average	sd	AOAA+IRINOTECAN average	sd
albumin	ALB	2,5 - 3,0 G/DL	2.53	0.50	2.73	0.49	2.67	0.42	2.25	0.21
alkaline phosphatase	ALP	35-96 U/L	53.75	23.68	45.67	6.03	47.33	3.79	39.25	8.38
alanine aminotransferase	ALT	17-77 U/L	39.25	3.40	32.33	5.51	30.33	1.53	34.50	15.02
amylase	AMY	U/L	898.00	175.58	714.67	41.55	716.00	98.06	766.75	114.93
total bilirubin	TBIL	0,0-0,9 MG/DL	0.38	0.05	0.50	0.26	0.33	0.06	0.35	0.06
urea nitrogen	BUN	8 - 33 MG/DL	21.00	1.63	22.00	5.57	23.33	4.73	19.00	2.16
calcium	CA	7,1-10,1 MG/DL	7.55	2.37	8.33	1.10	8.23	0.98	7.48	0.85
phosphorus	PHOS	5,7-9,2 MG/DL	5.23	0.57	4.30	0.72	4.77	0.50	4.80	0.41
creatinine	CRE	0,2-0,9 MG/DL	0.18	0.10	0.30	0.10	0.17	0.12	0.18	0.10
glucose	GLU	62-175 MG/DL	184.00	17.11	143.00	29.51	161.33	34.43	151.50	18.38
sodium	NA+	140-160 MMOL/L	154.25	18.01	145.33	4.04	149.33	2.08	146.50	2.52
potasium	K+	5,0-7,5 MMOL/L	5.20	0.59	5.20	0.69	5.23	0.40	5.05	0.47
total protein	TP	3,5-7,2 G/DL	4.48	0.67	4.53	0.61	4.60	0.70	4.23	0.48
globulin	BGLOB	G/DL	1.93	0.76	1.80	0.26	1.93	0.29	1.93	0.50

Supplementary Table 1. Comprehensive diagnostic blood profile analysis showed no signs of additional organ injury in mice treated with AOAA/irinotecan.
